# Impact of current or past physical activity level on functional capacities and body composition among elderly people: a cross-sectional analysis from the YMCA study

**DOI:** 10.1186/s13690-021-00573-9

**Published:** 2021-04-15

**Authors:** Fanny Buckinx, Éva Peyrusqué, Jordan Granet, Mylène Aubertin-Leheudre

**Affiliations:** 1 Département des Sciences de l’Activité Physique, Groupe de Recherche en Activité Physique Adapté, Université du Québec à Montréal (UQAM), Montréal, Qc Canada; 2 Centre de Recherche de l’Institut Universitaire de Gériatrie de Montréal (CRIUGM), Montréal, Qc Canada; 3 WHO Collaborating Centre for Public Health Aspects of Musculoskeletal Health and Ageing, University of Liège, Liège, Belgium

**Keywords:** Exercise, Physical performance, Muscle function, Muscle strength, aging, Lifestyle habits, Fitness center

## Abstract

**Background:**

Physical activity (PA) is recognized as important predictor of healthy aging. However, the influence of the type of voluntary PA as well as age or sex in this relationship is unclear. Thus, we assess the association between current and past PA level and physical performances among voluntary active older adults.

**Methods:**

Functional capacities (timed Up and Go, sit-to-stand, alternate step test, unipodal balance, grip strength, knee extension strength, estimated muscle power and VO2 max) as well as body composition (DXA: total and appendicular lean masses (LM; kg), fat mass (FM; %)) were measured. Current and last 5-years PA level (time spent on total, aerobic, resistance and body & mind activities) were assessed using an interview. Multiple regressions, adjusted on age, sex and BMI, were performed to assess the relationship between current or past PA level and physical performances. Sub-group analysis, according to the sex (men/women) or age (< 65 yrs. vs. ≥65 yrs) were performed.

**Results:**

525 subjects (age:61.7 ± 8.1 yrs.; women:68.9%; BMI:26.4 ± 4.8 kg/m^2^) were enrolled in this study. After adjustment on confounding factors, total current PA level has positive impact on total FM (β = − 2.09, *p* = 0004) and balance (β = 0.10; *p* = 0.05). Moreover, current body & mind activities influence total LBM (β = − 0.22, *p* = 0.02) and balance (β = 0.17; *p* = 0.001) whereas resistance activities influence total LBM (β = 0.17; *p* = 0.05), FM (β = − 0.16; *p* = 0.04) and sit-to-stand capacities (β = − 0.10; *p =* 0.05). Globally, these results were more pronounced in women than in men and among people aged over 65 years. Past level of PA has low impact on functional capacities and body composition, regardless of sex. Among people < 65 years, there is no relationship between time spent on total PA and functional capacities or body composition. However, a significant correlation was found between past total PA and balance (r=*`* 0.19; *p* = 0.01), alternate-step test (*r =* 0.24; *p* = 0.02) and VO2max (*r =* 0.19; *p =* 0.02) in people aged over 65 years. More precisely, the past time spent on aerobic and resistance activities influence balance (*r =* 0.16; *p* = 0.03 and *r =* 0.15; *p* = 0.04, respectively) after 65 yrs. old.

**Conclusion:**

Even if physical activity history has little influence on physical aging process, being active is associated with body composition and functional capacities, especially among women aged 65 years and over.

## Background

It is well established that the aging process is accompanied by decline in physical function and quality of life [1–3]. In particular, with advancing age a deterioration of physical function (i.e. aerobic capacities, muscle function and postural balance) is observed and is leading to impaired abilities to perform activities of daily living (ADL) [4]. Moreover, the decline in functional capacity of an older person is a powerful predictor of negative events, independent of the presence and number of disease conditions [5]. Therefore, the functional status has been proposed to be a critical target for interventions to restore robustness, improve quality of life and extend survival in late life [6]. In this sense, preservation of physical function is a major public health concern, where the role and importance of physical activity (PA) practice is recognized by world health organization [7]. Indeed, the deterioration in functional abilities observed with age [8] is accentuated by physical inactivity and sedentary lifestyle which affects 50% of the elderly [9]. In addition, this deterioration decreases the mobility of the elderly, creating a vicious cycle of deconditioning [9], which accelerates the spiral of loss of autonomy and increases the need for health care and services and therefore health costs [10]. Effectively, a large number of epidemiological research confirms the benefits of physical activity in reducing risk of various age-related morbidities and all-cause mortality [11]. More specifically, cross-sectional and longitudinal data demonstrate that 1) cardiorespiratory fitness is associated with functional capacity and independence; 2) strength and power are related to the performance and activities of daily living and; 3) Balance-mobility in combination with power are important factors in preventing falls [11]. Landi et al. also support that physical activity has an independent effect on functional autonomy among frail and older people [12]. In addition, two reviews of the literature assessing the association between physical activity and physical function have shown that a physically active lifestyle reduces the risk of low physical function among older adults [13, 14]. The recent umbrella review of Dipietro also reported that physical activity reduced the risk of fall-related injuries by 32 to 40%, including severe falls requiring medical care or hospitalization [15]. The authors have shown that physical activity improved physical function and reduced the risk of age-related loss of physical function. In addition, according to Dipietro et al., aerobic, muscle-strengthening, and/or multicomponent physical activity programs elicited the largest improvements in physical function in these same populations [15]. A systematic review of meta-analysis published in 2021 concluded also that resistance training and nutritional supplementation significantly improved muscle strength while supervised multimodal exercises and body vibration significantly improved balance and reduced the risk of falling [16]. Physical activity and more specifically increasing total PA and practicing moderate-to-vigorous PA is associated with improvements on body composition in older adults (55–75 years old) with overweight or obesity, and metabolic syndrome [17]. Consequently, being active and having history of PA habits are nowadays recognized as important predictors to prevent physical aging [7]. Evidence has shown that physical activity is strongly associated with healthier ageing trajectories [18, 19]. Exercise, or physical activity, is an important component of healthy aging, preventing or mitigating falls, pain, sarcopenia, osteoporosis, and cognitive impairment [20]. The meta-analysis of Daskalopoulou et al., which included 23 longitudinal studies assessing the associations between physical activity and healthy aging, suggested that physically active older adults (defined by any levels of exercises or activities) had almost 40% increased odds of experiencing healthy aging compared to their non-physically active counterparts [21]. The Moreno-Agostino’ findings also suggest a positive impact of physical activity on healthy ageing, attenuating declines in health and functioning [22]. However, it is unclear if sex or age influence this relationship and if a sub-type of voluntary PA is more efficient to maintain healthy aging.

Therefore, this study aimed to assess the impact of the last 5-years of PA level and functional capacities and body composition among elderly people. The secondary objectives of the present study are to assess this impact, according to sub-type of voluntary PA (i.e. aerobic, body & mind and resistance activities) but also according to sex and age of the subjects.

## Methods

### Study design and participants

This is a cross-sectional secondary study of the YMCA study. The YMCA study was performed in volunteer members aged over 50 years old from 5 ‘YMCA’ fitness center located in Montreal area. The baseline participant evaluation were performed from September 2010 to April 2012 were recruited. The prevalence of participants recruited in each YMCA center is relative to the number of registered members. In addition, the 5 YMCA have been chosen to include all socio-economic categories (area with low to high income): YMCA Center 1 (mean age: 62.6 ± 8.9 yrs.; *n* = 62 (12%), Women: 48 & Men: 14; Caucasian: 88.7%; at least college level:74.2%; living in couple: 62.9%); YMCA Center 2 (mean age: 62.0 ± 8.7 yrs.; *n* = 172 (33%), Women: 122 & Men: 50; Caucasian: 88.9%; at least college level:84.2%; living in couple: 51.2%); YMCA Center 3 (mean age: 63.0 ± 7.2 yrs.; *n* = 116 (22%), Women: 84 & Men: 32; Caucasian: 94.0%; at least college level:90.4%; living in couple: 62.0%); YMCA Center 4 (mean age: 59.5 ± 7.2 yrs.; *n* = 130 (25%), Women: 70 & Men: 60; Caucasian: 96.9%; at least college level:89.2%; living in couple: 51,6%); YMCA Center 5 (mean age: 61.8 ± 7.5 yrs.; *n* = 45 (9%),Women: 37 & Men: 8; Caucasian: 97.8%; at least college level:71.1%; living in couple: 31.3%); Average all YMCA center: (*n* = 525, women:361 (68,8%)/ men: 164; Caucasian: 92,7%; mean age: 61.7 ± 8.0 yrs.; at least college level:84.5%; living in couple: 53.3%).Baseline participants were recruited between september 2010 to april 2012 (18 months).

To be included in the study, participants had to (a) be age 50 yrs. old and over, (b) be registered as YMCA physical activity members, (c) be volunteer to participate in this investigation (d) live in the community, (e) understand and speak French or English. Participants were excluded if they answered “yes” to one or more of the 7 questions on the Physical Activity Readiness Questionnaire (PAR-Q) [23] or if they used walking aid.

Face-to-face interview and assessments were conducted. More specifically, after screening for the aforementioned inclusion criteria, participants were invited, every year and during 3 years, to visit their YMCA center where their muscle function, physical performance and functional capacity were assessed. They were also invited for an optional visit to the Department of Sciences de l’Activité Physique at the University of Quebec at Montreal, where their body composition was evaluated using Dual X-ray Absorptiometry (DXA). Because a significant number of people in this age group have already a bone density test as part as their regular medical monitoring, DXA measurements were recommended but not mandatory to avoid additional radiation.

The study was approved by the ethical committee of the institution (UQAM) under number A-109987. All participants were fully informed about the nature, goal, procedures, and risks of the study and gave their informed consent.

### Measurements

#### Level of physical activity

A structured interview conducted by a trained kinesiologist, was performed to determine the current and the last 5-years past physical activity levels using a grid including the list of activities available at the YMCAs and blank spaces to record unlisted activities,. The objective of the structured interview is to provide data regarding the duration, the frequency and the distribution over the week of low, moderate or vigorous activities, as well as the type of activity performed. Participants were asked to specify the practice time (in minutes/week) for each activity in which they were currently engaged, either inside or outside the YMCAs, and for how long these physical activities have been practiced (in months). Physical activities were then categorized into 3 main categories; Resistance training, Aerobic training and Body & mind practices. Based on this information, we were able to calculate the weekly time of physical practice, as well as the average duration of physical practices in total or by category of physical activity.

#### Physical performance

Different validated tests were used to assess functional capacities, muscle function and aerobic capacity:

##### Timed up and go” test

Walking speed was estimated using the “Timed Up & Go” test (in seconds). This test, which consists in standing from a chair, walking a 3-m distance and sitting down again [24], was performed at comfortable paced (TUG-n) and at a fast-paced (TUG-f) walking speed. A duration above 30 s indicates limited mobility and an increased risk of falling whereas a duration of less than 20 s indicates appropriate mobility with subject likely to be independent in activities of daily living [25]. Excellent reliability was observed for the TUG. ICC values for intra reliability were ranged between 0.97 and 0.99 while ICC values for inter reliability were ranged between 0.95 and 0.96 [26].

##### Balance

Participants were asked to stand on one leg for as long as possible (max: 60 s) with the arms along the side of the body. The test was interrupted after 60 s (maximum time) or if the legs touched each other, the feet moved on the floor or the swing leg touched the floor. The measure was repeated with the right and left leg (3 attempts for each leg), with the eyes opened. Best scores for right and left leg were recorded and averaged.

##### Alternate step test

Weight shifting ability in the forward and upward directions was estimated using the alternate-step test. Participants were placed facing toward a 20-cm height step and instructed to touch its top with the right and left foot, alternatively, as fast as possible during a 20-s period [27]. The number of steps was recorded for analysis. Excellent inter-rater and intra-rater reliability was observed for the Alternate-step-test (ICC > 0.9) [27].

##### “Sit-to-stand” test

Lower-body function was measured using the “sit-to stand” test. Subjects were asked to stand up from a standard height (45 cm) chair and to sit down 10 times as fast as possible, with arms across their chests [28]. The time (in seconds) to perform the task was recorded. This test showed good test-retest reliability (i.e. Intraclass Correlation Coefficient: ICC = 0.94–0.99) [29].

##### Muscle power

Based on the functional sit-to-stand test, a power index was also calculated using the Takaï equation, which has been shown to be reliable, accurate and validated in older adults (see the equation below) [30]. Short-term reliability of lower limb muscle power is very high with ICC ranged between 0.88 and 0.96. Long-term reliability, was also very high with ICC ranged between 0.94 and 0.96) [31].

Takaï equation: $$ \mathrm{P}=\frac{\left(\mathrm{L}-\mathrm{A}\right)\times \mathrm{BM}\times \mathrm{g}\times 10}{\mathrm{T}} $$

*Where P ≡ Power (watts); L ≡ Leg length (the distance from the greater trochanter of the femur to the lateral malleolus) (m); A ≡ Height of the chair (m); BM ≡ Body mass; g ≡ Acceleration due to gravity (9.8 m∙s-2); 10 ≡ number of repetitions; T ≡ Time to perform the test, respectively.*

##### Grip strength

Maximum voluntary upper limb muscle strength was measured (in kg) using a hand dynamometer with adjustable grip (Lafayette Instrument). Participants were standing upright with the arm along the side of the body with the elbow extended and the palm of the hand facing the thigh. Participants were advised to squeeze as hard as possible the hand dynamometer for up to 4 s. Three measurements for each hand, alternatively, were performed and the maximal score for each was recorded [32]. According to the systematic review of Bohannon (2017), test-retest reliability of grip strength measures obtained by dynamometry was good to excellent (intra-class correlation coefficients > 0.80) [33].

- Knee extension strength (KES): Participants’ right leg’s maximum KES was determined by one repetition maximum (1RM) on a standard Atlantis C-105 knee extension machine and expressed in kilograms (kg). After a five –repetition warm-up, a resistance was chosen that was estimated to be slightly below the subject’s 1RM value. If the subject was able to complete the repetition, the resistance was increased by 5 kg and another trial performed after a 60-s rest. Each participant was given six lifting attempts in order to achieve their 1RM. A repetition was valid if the participant used correct form and was able to complete the entire lift in a controlled manner without assistance. The last resistance that was successfully completed was recorded as the 1RM [34].

##### Estimated maximal oxygen consumption

The maximal oxygen consumption (VO2max) was estimated using the 2-km walk test (UKK Institute, Tampere, Finland). This test has been shown to be reliable and valid in an older population [35]. Participants were asked to walk a 2-kmdistance at a brisk pace on an indoor 100-m track. Heart rate was monitored during the whole walk using a Polar system (S810, Oy, Finland). VO2max (ml·kg-1·min-1) was estimated using gender-specific equations as previously described [35].

#### Body composition

Body weight (BW) was measured once using an electronic scale (Tanita BC-558, USA). Height was measured once using a stadiometer (Seca, Hanover, MD) affixed to the wall. Body mass index [BMI = Body weight (kg)/Height (m^2^)] was then calculated.

Dual-energy X-ray absoptiometry [DXA] (GE Prodigy Lunar Corporation version 6.10.019, Madison, USA) was used to assess body composition and more specifically fat mass (%) and lean mass (kg) but also to determine appendicular lean mass index (kg/m^2^). Participants were required to remove all jewels prior to being positioned on the DXA table. Scans were performed with subjects in a supine position. The entire body was scanned beginning at the top of the head and moving in a rectilinear pattern down the body to the feet. The regions of the body were automatically determined by the system ROIs [36]. The coefficient of variation of this device, in vivo, is 0.8% for fat percentage, 2.1% for total fat mass, and 1.5% for total lean mass. The DXA has been regularly calibrated throughout the study (i.e. every week) and a specialized technician carried out measurements.

### Statistical analysis

Data distribution was tested using the Kolmogorov test. Continuous variables were expressed as mean ± standard deviation (SD). Descriptive variables were expressed as a percentage. An independent sample t-test was used to identify between-group baseline differences. The Chi-squared test or Fisher test were used to compare frequency of observations between groups.

Multiple regressions, adjusted for age, sex and BMI, were performed to assess the relationship between current or past physical activity level and body composition or functional capacities. Subgroup analysis, according to the sex (man vs. women) and age (< 65y vs. ≥ 65y) were also performed by means of Pearson Correlations. All statistical analyses were performed using SPSS 25.0 (Chicago, IL, USA). *p* ≤ 0.05 was considered statistically significant.

## Results

### Population

A total of 525 volunteers subjects were enrolled in this study. The mean age of the participants was 61.7 ± 8.1 years and 68.9% of them were women. They had a mean BMI of 26.4 ± 4.8 kg/m^2^.

### Impact of current PA level or type of PA on functional capacities and body composition

After adjustment on confounding factors, total current physical activity level has positive impact on total fat mass (β = − 2.09, *p* = 0004) and balance (β = 0.10; *p* = 0.05). Moreover, current body & mind activities influence total LBM (β = − 0.22; *p* = 0.02) and balance (β = 0.17; *p* = 0.001) whereas current resistance activities influence total LBM (β = 0.17; *p* = 0.05), total fat mass (β = − 0.16; *p* = 0.04) and sit-to-stand test (β = − 0.10; *p =* 0.05) (Table 1).

**Table 1:** Impact of current PA level or type of PA on functional capacities and body composition

	Total PA level (min)		Body & mind activities (min)		Aerobic activities (min)		Resistance activities (min)	
	β	p	Β	P	β	p	β	p
**BODY COMPOSITION**								
Total LBM (kg)	0.13	0.14	−0.22	**0.02**	0.16	0.09	0.17	**0.05**
Appendicular lean mass (kg/m^2^)	0.14	0.17	−0.05	0.94	0.13	0.14	0.10	0.25
Total Fat mass (%)	−2.09	**0.04**	0.05	0.57	−0.15	0.07	− 0.16	**0.04**
**MUSCLE FUNCTION**								
Grip strength (kg)	−0.23	0.82	−0.04	0.49	0.02	0.73	−0.08	0.24
Relative grip strength (kg/BW)	−0.21	0.5	0.06	0.86	−0.21	0.49	−0.21	0.49
Muscle Power (N; Takaï)	0.07	0.20	−0.03	0.58	0.08	0.16	0.04	0.55
Knee extension strength (kg)	−0.03	0.59	−0.04	0.48	0.06	0.36	0.11	0.09
**FUNCTIONAL CAPACITIES**								
TUG (s)	0.01	0.83	0.001	0.98	−0.02	0.69	0.02	0.67
Fast TUG (s)	−0.08	0.08	−0.02	0.71	0.005	0.92	−0.02	0.66
Sit-to-stand (s)	−0.08	0.13	0.04	0.43	−0.07	0.18	−0.10	**0.05**
Balance (s)	0.10	**0.05**	0.17	**0.001**	0.05	0.34	0.03	0.58
Alternate step test (n)	0.05	0.34	0.05	0.33	0.06	0.20	−0.08	0.10
**AEROBIC CAPACITIES**								
Estimated VO2 max (ml/min/kg)	0.06	0.58	0.18	0.10	−0.04	0.72	0.15	0.16

*Legend*: *Multiple regression adjusted on age, sex and BMI; Significant: P < 0.05. TUG* timed up and Go, *LBM* lean body mass

#### According to the sex of the participants

Sub-analysis shows that total current physical activity level was significantly associated with total fat mass (*r =* − 0.17; *p* = 0.001), sit-to-stand test (*r =* − 0.16; *p* = 0.007), balance (*r =* 0.13; *p* = 0.01) and estimated VO2 max (*r =* 0.11; *p* = 0.04) in women (*n* = 362) but not in men (*n* = 163) (Tables 2 and 3). More specifically, current Body & Mind activities are correlated with total LBM (*r =* − 0.14; *p* = 0.008), appendicular lean mass (*r =* − 0.13; *p* = 0.02) and balance (*r =* 0.12; *p =* 0.02) in women (Table 3*).* Then, current aerobic activities are associated with total LBM (*r =* 0.011; *p* = 0.04), total fat mass (*r =* − 0.13; *p* = 0.015) and sit-to-stand (*r =* − 0016; *p =* 0.008) in women (Table 3). Finally, current resistance activities are associated with appendicular lean mass (*r =* − 0.11; *p* = 0.04), total fat mass (*r =* 0.16; *p* = 0.02), sit-to stand (β-*r =* − 0.12; *p* = 0.05) and estimated Vo2 max (*r =* 0.12; *p* = 0.03) in women (Table 3) but also knee extension strength (*r =* 0.16; *p =* 0.04) and alternate step test (*r =* − 0.16; *p =* 0.04) in men (Table 2).

**Table 2:** Correlations between current PA level or type of PA and functional capacties and body composition, among men (*n* = 163)

	Total PA level (min)		Body & mind activities (min)		Aerobic activities (min)		Resistance activities (min)	
	R	p	r	P	R	p	r	p
**BODY COMPOSITION**								
Total LBM (kg)	0.16	0.84	0.11	0.17	0.02	0.81	0.08	0.30
Appendicular lean mass (kg/m^2^)	0.05	0.52	0.06	0.45	0.04	0.59	−0.003	0.97
Total Fat mass (%)	0.06	0.49	0.03	0.74	0.03	0.74	−0.08	0.31
**MUSCLE FUNCTION**								
Grip strength (kg)	0.01	0.94	0.03	0.73	0.04	0.63	−0.06	0.44
Relative grip strength (kg/BW)	−0.65	0.16	−0.78	0.07	−0.44	0.38	−0.76	0.08
Muscle Power (N; Takaï)	0.07	0.48	−0.05	0.61	0.07	0.51	0.11	0.28
Knee extension strength (kg)	0.018	0.83	−0.07	0.39	−0.019	0.82	0.16	**0.04**
**FUNCTIONAL CAPACITIES**								
TUG (s)	0.09	0.25	0.07	0.41	−0.09	0.22	0.026	0.74
Fast TUG (s)	−0.05	0.55	−0.014	0.86	−0.08	0.35	0.04	0.63
Sit-to-stand (s)	−0.06	0.57	0.013	0.89	0.03	0.75	−0.13	0.20
Balance (s)	0.13	0.09	0.09	0.27	0.11	0.15	0.05	0.56
Alternate step test (n)	0.04	0.63	0.06	0.46	0.09	0.26	−0.16	**0.04**
**AEROBIC CAPACITIES**								
Estimated VO2 max (ml/min/kg)	0.07	0.42	−0.05	0.47	0.11	0.20	−0.03	0.71

*Legend: Pearson correlations; Significant: P < 0.05. TUG* timed up and Go*, LBM lean body mass*

**Table 3:** Correlations between current PA level or type of PA and functional capacities and body composition, among women (*n =* 362)

	Total PA level (min)		Body & mind activities (min)		Aerobic activities (min)		Resistance activities (min)	
	r	P	r	P	r	p	r	p
**BODY COMPOSITION**								
Total LBM (kg)	0.04	0.46	−0.14	**0.008**	0.11	**0.04**	−0.06	0.26
Appendicular lean mass (kg/m^2^)	−0.01	0.83	−0.13	**0.02**	0.06	0.26	−0.11	**0.04**
Total Fat mass (%)	−0.17	**0.001**	−0.01	0.82	−0.13	**0.015**	0.16	**0.02**
**MUSCLE FUNCTION**								
Grip strength (kg)	0.17	0.75	−0.06	0.26	0.05	0.33	−0.05	0.38
Relative grip strength (kg/BW)	0.21	0.23	0.23	0.19	0.15	0.39	−0.02	0.91
Muscle Power (N; Takaï)	0.07	0.29	−0.03	0.61	0.09	0.16	−0.02	0.74
Knee extension strength (kg)	0.004	0.95	−0.04	0.39	0.02	0.74	0.002	0.98
**FUNCTIONAL CAPACITIES**								
TUG (s)	−0.019	0.72	0.03	0.52	−0.02	0.64	−0.02	0.66
Fast TUG (s)	−0.043	0.42	−0.01	0.79	−0.02	0.76	−0.09	0.11
Sit-to-stand (s)	−0.16	**0.007**	0.07	0.27	−0.16	**0.008**	−0.12	**0.05**
Balance (s)	0.13	**0.01**	0.12	**0.02**	0.08	0.11	0.06	0.28
Alternate step test (n)	0.09	0.07	0.02	0.68	0.09	0.08	0.011	0.84
**AEROBIC CAPACITIES**								
Estimated VO2 max (ml/min/kg)	0.11	**0.04**	0.05	0.41	0.07	0.21	0.12	**0.03**

*Legend: Pearson correlations; Significant: P < 0.05. TUG* timed up and Go, *LBM* lean body mass

#### According to the age of the participants

Subjects were divided in two groups: < 65 years (*n* = 338) and ≥ 65 years (*n* = 186). Among people under 65 years old, total current PA level is correlated with total fat mass (*r =* − 0.13; *p =* 0.02) and balance (*r =* 0.12; *p* = 0.03) (Table 4). In this sub-group, the current time spent on body & mind activities influences total LBM (*r =* − 0.18; *p* = 0.001), appendicular lean mass (*r =* − 0.17; *p =* 0.001); grip strength (*r =* − 0.12; *p* = 0.02), knee extension strength (*r =* − 0.16; *p* = 0.006) and balance (*r =* 0.14; *p* = 0.01) while the current time spent on resistance activities influence fat mass (*r =* − 0.14; *p =* 0.001) (Table 4). However, the current time spent on aerobic activities does not affect body composition, muscle strength, functional capacities and aerobic capacities in subjects < 65 years (Table 4).

**Table 4:** Correlations between current PA level or type of PA and functional capacities and body composition, among subjects< 65 years old (*n =* 338)

	Total PA level (min)		Body & mind activities (min)		Aerobic activities (min)		Resistance activities (min)	
	r	p	r	p	r	p	r	p
**BODY COMPOSITION**								
Total LBM (kg)	0.001	0.98	−0.18	**0.001**	0.05	0.37	0.02	0.69
Appendicular lean mass (kg/m^2^)	−0.008	0.88	−0.17	**0.001**	0.05	0.41	0.004	0.94
Total fat mass (%)	−0.13	**0.02**	−0.02	0.72	−0.09	0.09	−0.14	**0.001**
**MUSCLE FUNCTION**								
Grip strength (kg)	0.02	0.61	−0.12	**0.02**	0.08	0.14	−0.03	0.58
Relative grip strength (kg/BW)	−0.15	0.44	0.11	0.56	−0.16	0.42	−0.23	0.24
Muscle Power (N; Takaï)	0.016	0.82	−0.12	0.08	0.07	0.29	−0.17	0.34
Knee extension strength (kg)	−0.03	0.57	−0.16	**0.006**	−0.016	0.78	0.09	0.12
**FUNCTIONAL CAPACITIES**								
TUG (s)	−0.04	0.49	−0.02	0.69	−0.06	0.29	0.07	0.20
Fast TUG (s)	− 0.03	0.64	0.002	0.97	−0.04	0.51	0.02	0.67
Sit-to-stand (s)	−0.06	0.36	0.06	0.40	−0.08	0.24	−0.03	0.65
Balance (s)	0.12	**0.03**	0.14	**0.01**	0.08	0.15	0.03	0.62
Alternate step test (n)	0.05	0.40	0.02	0.74	0.07	0.18	−0.09	0.09
**AEROBIC CAPACITIES**								
Estimated VO2 max (ml/min/kg)	0.06	0.29	0.07	0.21	0.03	0.64	0.05	0.34

*Legend: Pearson correlations; Significant: P < 0.05. TUG* timed up and Go, *LBM* lean body mass

Table 5 highlights that total current PA level is correlated with knee extension strength (*r =* 0.18; *p* = 0.02), sit-to-stand (*r =* − 0.19; *p =* 0.02) and VO2 max (*r =* 0.18; *p =* 0.02) among people aged 65 years and over. In this sub-group, the current time spent on body & mind activities is associated with total LBM (*r =* − 0.18; *p* = 0.01) and total fat mass (*r =* 0.24; *p =* 0.001) while the current time spent on aerobic activities influence VO2 max (*r =* 0.18; *p =* 0.02) (Table 5). Among people aged ≥65 years, the current time spent on resistance activities is significantly associated with body composition (i.e. total LBM *r =* 0.35; *p* < 0.001, appendicular lean mass *r =* 0.25; *p =* 0.001 and total fat mass *r =* − 0.31; *p <* 0.001); muscle strength (i.e. grip strength *r =* 0.25; *p =* 0.001, muscle power *r =* 0.22; *p =* 0.009, knee extension strength *r =* 0.33; *p <* 0.001) and functional capacities (i.e. fast TUG *r =* − 0.19; *p =* 0.01 and sit-to-stand *r =* − 0.23; *p* = 0.004) (Table 5).

**Table 5:** Correlations between current PA level or type of PA and functional capacities and body composition, among subjects≥65 years old (*n =* 186)

	Total PA level (min)		Body & mind activities (min)		Aerobic activities (min)		Resistance activities (min)	
	R	p	r	P	r	p	R	p
**BODY COMPOSITION**								
Total LBM (kg)	0.13	0.08	−0.18	**0.01**	0.07	0.34	0.35	**< 0.001**
Appendicular lean mass (kg/m^2^)	0.13	0.09	−0.02	0.84	0.06	0.45	0.25	**0.001**
Fat mass (%)	−0.12	0.12	0.24	**0.001**	−0.09	0.23	−0.31	**< 0.001**
**MUSCLE FUNCTION**								
Grip strength (kg)	0.04	0.57	−0.13	0.09	−0.005	0.95	0.25	**0.001**
Relative grip strength (kg/BW)	0.38	0.23	0.15	0.63	0.38	0.23	0.11	0.72
Muscle Power (N; Takaï)	0.15	0.08	0.02	0.81	0.09	0.30	0.22	**0.009**
Knee extension strength (kg)	0.18	**0.02**	−0.04	0.63	0.10	0.20	0.33	**< 0.001**
**FUNCTIONAL CAPACITIES**								
TUG (s)	−0.04	0.57	0.06	0.43	− 0.015	0.85	−0.14	0.06
Fast TUG (s)	−0.07	0.33	0.014	0.86	−0.02	0.80	−0.19	**0.01**
Sit-to-stand (s)	−0.19	**0.02**	0.05	0.57	− 0.14	0.08	−0.23	**0.004**
Balance (s)	0.14	0.06	0.09	0.20	0.08	0.29	0.11	0.13
Alternate step test (n)	0.11	0.16	0.05	0.51	0.09	0.24	0.03	0.67
**AEROBIC CAPACITIES**								
Estimated VO2 max (ml/min/kg)	0.18	**0.02**	−0.08	0.33	0.18	**0.02**	0.12	0.14

*Legend: Pearson correlations; Significant: P < 0.05. TUG* timed up and Go*, LBM* lean body mass

### Impact of past PA level or type of PA on functional capacities and body composition

As shown in Table 6, after adjustment on confounding factors, past type of level of PA (last 5 years) has no impact on functional capacities and body composition.

**Table 6:** Impact of past PA level or type of PA on functional capacities and body composition

	Total PA level (min)		Body & Mind activities (min)		Aerobic activities (min)		Resistance activities (min)	
	β	p	β	p	β	p	β	p
**BODY COMPOSITION**								
Total LBM (kg)	−0.007	0.94	0.08	0.40	−0.05	0.57	−0.12	0.17
Appendicular lean mass (kg/m^2^)	−0.11	0.23	0.07	0.40	−0.001	0.99	−0.017	0.06
Total Fat mass (%)	0.04	0.67	−0.06	0.47	0.10	0.19	−0.09	0.29
**MUSCLE FUNCTION**								
Grip strength (kg)	−0.05	0.48	0.06	0.36	0.01	0.86	−0.08	0.23
Relative grip strength (kg/BW)	−0.23	0.45	−0.04	0.91	−0.40	0.19	0.05	0.87
Muscle Power (N; takaï)	0.02	0.75	0.04	0.46	−0.008	0.89	−0.07	0.24
Knee extension strength (kg)	−0.2	0.74	0.018	0.77	0.09	0.14	−0.06	0.31
**FUNCTIONAL CAPACITIES**								
TUG (s)	0.07	0.14	−0.02	0.74	−0.02	0.73	0.02	0.68
Fast TUG (s)	0.006	0.12	−0.09	0.08	0.02	0.64	−0.06	0.19
Sit-to-stand (s)	−0.05	0.35	−0.001	0.99	−0.02	0.69	0.06	0.28
Balance (s)	0.05	0.39	0.05	0.38	0.10	0.05	0.09	0.09
Alternate step test (n)	0.04	0.48	−0.006	0.90	−0.04	0.43	−0.08	0.10
**AEROBIC CAPACITIES**								
Estimated VO2 max (ml/min/kg)	0.009	0.86	− 0.045	0.38	0.04	0.89	0.018	0.72

*Legend: Multiple regression adjusted on age, sex and BMI*. *Significant: P < 0.05. TUG* timed up and Go, *LBM* lean body mass

#### According to the sex of the participants

After adjustment on confounding factors, past level of PA or type of PA were not correlated with body composition, muscle strength, functional capacities and aerobic capacities in men (*n* = 163) (Table 7).

**Table 7:** Correlations between past PA level or type of PA on functional capacities and body composition, among men (*n =* 163)

	Total PA level (min)		Body & mind activities (min)		Aerobic activities (min)		Resistance activities (min)	
	r	p	r	p	r	p	r	p
**BODY COMPOSITION**								
Total LBM (kg)	0.002	0.98	0.07	0.36	− 0.13	0.10	− 0.03	0.75
Appendicular lean mass (kg/m^2^)	0.05	0.57	0.05	0.53	−0.11	0.18	−0.05	0.54
Total Fat mass (%)	0.01	0.89	−0.14	0.08	−0.006	0.94	0.07	0.36
**MUSCLE FUNCTION**								
Grip strength (kg)	−0.006	0.94	0.06	0.47	−0.006	0.94	0.005	0.95
Relative grip strength (kg/BW)	0.011	0.98	0.06	0.98	−0.14	0.79	0.19	0.72
Muscle Power (N; Takaï)	0.11	0.31	0.13	0.21	−0.04	0.74	−0.08	0.47
Knee extension strength (kg)	0.12	0.19	0.12	0.16	0.16	0.06	−0.08	0.34
**FUNCTIONAL CAPACITIES**								
TUG (s)	0.015	0.86	−0.07	0.38	−0.08	0.32	0.07	0.39
Fast TUG (s)	0.05	0.49	−0.04	0.64	−0.006	0.94	−0.02	0.77
Sit-to-stand (s)	−0.13	0.19	−0.05	0.65	−0.12	0.25	0.08	0.46
Balance (s)	0.009	0.92	0.02	0.77	0.09	0.23	0.04	0.65
Alternate step test (n)	0.13	0.11	0.07	0.37	0.08	0.34	0.05	0.51
**AEROBIC CAPACITIES**								
Estimated VO2 max (ml/min/kg)	0.05	0.58	−0.07	0.37	0.03	0.75	0.008	0.92

*Legend: Pearson correlations; Significant: P < 0.05. TUG* timed up and Go, *LBM* lean body mass

Among women, we observed a significant relationship between past PA level and balance (*r =* 0.015; *p* = 0.005) (Table 8). In addition, past resistance activities influence appendicular lean mass (*r =* − 0.11; *p* = 0.04) and alternate step test (*r =* − 0.12; *p* = 0.02) while past body & mind activities or past aerobic activities has no influence on studied parameters (Table 8).

**Table 8:** Correlations between past PA level or type of PA and functional capacities and body composition, among women (*n =* 362)

	Total PA level (min)		Body & mind activities (min)		Aerobic activities (min)		Resistance activities (min)	
	r	p	r	p	r	p	r	p
**BODY COMPOSITION**								
Total LBM (kg)	0.02	0.68	−0.07	0.18	0.04	0.48	0.09	0.07
Appendicular lean mass (kg/m^2^)	0.05	0.35	−0.06	0.27	0.06	0.29	−0.11	**0.04**
Total Fat mass (%)	−0.06	0.29	−0.02	0.73	0.02	0.69	−0.10	0.06
**MUSCLE FUNCTION**								
Grip strength (kg)	0.05	0.39	0.01	0.84	0.03	0.56	−0.09	0.08
Relative grip strength (kg/BW)	0.06	0.74	−0.03	0.86	−0.29	0.08	0.12	0.50
Muscle Power (N; Takaï)	0.015	0.80	−0.02	0.75	0.011	0.86	− 0.07	0.28
Knee extension strength (kg)	0.02	0.68	−0.08	0.18	0.03	0.55	−0.03	0.55
**FUNCTIONAL CAPACITIES**								
TUG (s)	0.04	0.48	0.007	0.90	−0.011	0.84	−0.011	0.83
Fast TUG (s)	−0.09	0.11	−0.09	0.08	0.003	0.95	−0.07	0.19
Sit-to-stand (s)	−0.06	0.33	0.04	0.49	−0.02	0.79	0.05	0.39
Balance (s)	0.015	**0.005**	0.05	0.33	0.09	0.07	0.08	0.12
Alternate step test (n)	0.06	0.29	−0.04	0.47	−0.06	0.28	−0.12	**0.02**
**AEROBIC CAPACITIES**								
Estimated VO2 max (ml/min/kg)	0.04	0.81	−0.16	0.78	0.014	0.79	0.04	0.52

*Legend: Pearson correlations; Significant: P < 0.05. TUG* timed up and Go, *LBM* lean body mass

#### According to the age of the participants

Subjects were divided in two groups: < 65 years (*n* = 338) and ≥ 65 years (*n* = 186). Among subjects aged less than 65 years old, we observed no relationship between past time spent on total physical activity and body composition, muscle strength, functional and aerobic capacities (Table 9). We observed a significant relationship between past time spent in aerobic activities and total LBM (*r =* − 0.10; *p* = 0.05) but also between past time spent in resistance activities and sit-to-stand (*r =* 0.15, *p =* 0.02) or alternate step test (*r =* 0.15; *p* = 0.007) (Table 9).

**Table 9:** Correlations between past PA level or type of PA on functional capacities and body composition, among subjects< 65 years old (*n =* 338)

	Total PA level (min)		Body & mind activities (min)		Aerobic activities (min)		Resistance activities (min)	
	r	p	R	p	r	p	r	p
**BODY COMPOSITION**								
Total LBM (kg)	0.003	0.57	0.009	0.86	−0.10	**0.05**	−0.07	0.23
Appendicular lean mass (kg/m^2^)	−0.011	0.84	0.01	0.86	−0.11	0.05	−0.07	0.21
Total Fat mass (%)	−0.02	0.74	−0.08	0.13	0.08	0.14	−0.05	0.42
**MUSCLE FUNCTION**								
Grip strength (kg)	−0.04	0.48	0.04	0.51	−0.08	0.15	0.02	0.67
Relative grip strength (kg/BW)	0.15	0.43	0.07	0.72	−0.38	0.05	0.25	0.19
Muscle Power (N; Takaï)	0.07	0.31	0.05	0.48	−0.06	0.37	−0.11	0.11
Knee extension strength (kg)	−0.005	0.93	0.05	0.40	0.001	0.98	−0.08	0.19
**FUNCTIONAL CAPACITIES**								
TUG (s)	0.02	0.79	−0.06	0.30	−0.03	0.55	−0.02	0.72
Fast TUG (s)	0.001	0.99	−0.01	0.82	0.04	0.48	−0.03	0.55
Sit-to-stand (s)	−0.04	0.53	0.06	0.39	0.002	0.98	0.15	**0.02**
Balance (s)	0.008	0.81	0.03	0.65	0.04	0.42	0.03	0.65
Alternate step test (n)	−0.01	0.14	−0.05	0.37	−0.08	0.17	0.15	**0.007**
**AEROBIC CAPACITIES**								
Estimated VO2 max (ml/min/kg)	0.04	0.48	−0.03	0.63	−0.008	0.88	−0.008	0.89

*Legend: Pearson correlations; Significant: P < 0.05. TUG* timed up and Go, *LBM* lean body mass

**Table 10:** Correlations between past PA level or type of PA on functional capacities and body composition, among subjects≥65 years old (*n =* 186)

	Total PA level (min)		Body & mind activities (min)		Aerobic activities (min)		Resistance activities (min)	
	r	p	R	P	r	p	r	P
**BODY COMPOSITION**								
Total LBM (kg)	0.04	0.62	−0.11	0.13	−0.10	0.17	−0.04	0.57
Appendicular lean mass (kg/m^2^)	0.08	0.31	−0.11	0.13	−0.06	0.43	−0.07	0.35
Total Fat mass (%)	0.004	0.96	0.09	0.23	0.08	0.31	0.006	0.93
**MUSCLE FUNCTION**								
Grip strength (kg)	0.08	0.31	−0.06	0.39	−0.03	0.45	−0.07	0.38
Relative grip strength (kg/BW)	0.20	0.53	−0.31	0.34	0.09	0.76	0.18	0.57
Muscle Power (N; Takaï)	−0.03	0.77	0.008	0.92	−0.04	0.62	−0.19	0.82
Knee extension strength (kg)	0.12	0.15	−0.11	0.17	0.01	0.89	−0.02	0.81
**FUNCTIONAL CAPACITIES**								
TUG (s)	0.12	0.13	0.02	0.82	−0.073	0.92	0.06	0.44
Fast TUG (s)	−0.05	0.55	−0.18	**0.01**	−0.01	0.88	−0.09	0.20
Sit-to-stand (s)	−0.10	0.20	−0.05	0.55	−0.06	0.44	−0.05	0.57
Balance (s)	0.19	**0.01**	0.10	0.18	0.16	**0.03**	0.15	**0.04**
Alternate step test (n)	0.24	**0.02**	0.11	0.13	0.07	0.38	0.09	0.23
**AEROBIC CAPACITIES**								
Estimated VO2 max (ml/min/kg)	0.19	**0.02**	−0.03	0.76	0.06	0.48	0.10	0.19

*Legend: Pearson correlations; Significant: P < 0.05. TUG* timed up and Go, *LBM* lean body mass

However, in subjects aged 65 years or older, we observed significant correlations between total past physical activity and balance (*r =* 0.19; *P* = 0.01), alternate-step test (*r =* 0.24; *P* = 0.02) or VO2 max (*r =* 0.19; *P =* 0.02). Moreover, in this group, we observed that the past time spent on aerobic or resistance activities influence balance (*r =* 0.16; *P* = 0.03 and *r =* 0.15; *P* = 0.04, respectively) (Table 10).

## Discussion

Our study assessing the association between current or past physical activity level and physical performance among YMCA members aged over 50 years old suggested that, after adjustment for confounding factors, total current physical activity level has positive impact on total fat mass and balance. More specifically, current body & mind activities influence total LBM and balance, whereas current resistance activities influence total LBM, total fat mass and sit-to-stand capacity. Globally, the results were more pronounced in women than in men and among people aged 65 years or older. Although methodological differences are observed between our research and previously published studies (i.e. methodology used to assess PA level: objective vs. self-report; physical function: dimensions covered), our results are in concordance with previously published data highlighted that lower level of PA level at present age was related to lower physical function in older adults [37–39]. This suggests that adopting a physically active lifestyle at old age has a positive effect on functional capacities. Our results are also in line with evidence suggesting that maintaining physical activity over time is key to preventing age-related body composition changes associated with aging [36, 40]. Regarding the sub-type of PA, our results confirm those published in the meta-analysis of Peterson et al. concluding that resistance trainings are effective for eliciting gains in lean body mass among older adults [41]. We also showed that resistance activities have a positive effect on fat mass and this had been suggested in a recent randomized controlled trial including 45 subjects (27 females, 18 males) aged between 65 and 75 years old from Murcia (Spain) [42]. In this study, participants receiving 12 weeks of moderate-to-high intensity resistance circuit training showed a significant increment of total lean body mass and a decrease of fat mass [42]. Then, our results demonstrated an improvement of sit-to-stand capacity among older adults participating in current resistance activities. Falhman et al. made the same observation in their quasi-experimental trial enrolling eighty-seven participants (65–93 years) living independently [43]. Furthermore, the observed relationship between current body & mind activities and balance is not surprising since a meta-analysis published in 2016 concluded that older adults who practiced body & mind activities not less than 150 min per week during at least 15 weeks could promote the balance ability [44]. Thus, body & mind movements (e.g., Tai Chi, yoga, Qigong, Pilates) may be a method for balance rehabilitation. We also observed a significant association between current body & mind activities and total lean body mass among older adults. This result is in line with a systematic review (meta-analysis) among which benefits of body & mind activities (i.e. yoga) may exceed those of conventional exercise interventions for self-rated health status, aerobic fitness, and strength [45]. Moreover, although more women than men do not meet the current guidelines on minimum level of PA per week [7], our results were more pronounced in women than in men. In fact, it is admitted that sex and gender differences exist in the benefits of different levels of physical activity specific to a range of poor health outcomes. For example, low to moderate intensity physical activity provides protection from cardiovascular disease [30] and diabetes to a greater extent among women [46] compared to men. However, few data comparing the effects of PA level on physical capacity and body composition between men and women exist in the literature. Finally, our results were more pronounced among people aged 65 years or older compared to young older adults. A possible explanation is that physical capacity was more likely to improve in subjects who had lower performance at baseline (i.e. among women compared to men; among older adults compared to young older adults), since the range of progression is greater. Mangine et al. recently highlighted that a physical activity program (i.e. a 8-week high-volume, moderate-intensity training program) stimulated adaptations in all participants, but the muscle strength improvements were significantly greater in weaker participants compared to stronger participants at baseline [47].

In the total population, our research demonstrated that past PA has limited impact on body composition and functional capacities, mainly in men. Among women, we observed a significant relationship between past PA level and balance. More specifically, past resistance activities influence appendicular lean mass and alternate step test in this group. However, Stenholm et al. demonstrated in the InCHIANTI cohort (1149 subjects aged 65 years and over) that higher cumulative physical activity over the life course was associated with less decline in physical performance in older ages [48]. Our results are consistent with the InCHIANTI study since past level of PA seems to influence functional capacities among people aged ≥65 y but not among younger subjects. Physical activity across the life trajectory may relate to physical function in a number of ways. Most importantly, the direct effects of physical activity (depending on the type of activity) include improvements in muscle strength, aerobic fitness, flexibility or balance and this is what we observed in the present study.

Our results contribute to the development of health policies by showing the importance of being even at an advanced age. Our findings are consistent with the WHO report entitled « Decade of Healthy Aging (2020–2030) » since we suggest that the type of physical activity must be chosen according to the expected health benefits. More specifically, to improve balance and therefore reduce the risk of falls, body & mind activities should be privileged while resistance activities should be recommended to improve body composition (lean body mass and fat mass) and lower limb muscle strength.

Some limitations must be emphasized. First, the cross sectional design of the study could preclude causal interpretation. Then, the questionnaire related to the past level of PA could induce a recall bias. Because interview were conducted in face-to-face, a social desirability could also be present. Finally, compared to the general elderly population, our sample has a good health status (i.e. due to our selection criteria), which could affect external validity of the results. Future studies should confirm our findings by utilizing longitudinal data with repeated measurement of physical activity preferably based on objective measurements.

## Conclusion

In conclusion, being active is associated with body composition and functional capacities, especially among women aged 65 years and over. Nevertheless, short-term PA history (< 5 yrs) has little influence on functional capacities and body composition in healthy aging population (> 50 years). But among people over 65 years, these effects of past PA are promising and required a specific attention. Overall, the present study offers an interesting perspective, suggesting that promotion of increased time in PA should be a prioritized target in order to counteract age-related physical decline.

## Data Availability

The data analyzed in the current study are not publicly available due to restrictions based in the General Data Protection Regulation (GDPR) on sensitive data such as personal health data.

## Abbreviations

PA: Physical activity
DXA: Dual energy X-rays absorptiometry
LBM: Lean body mass
FM: Fat mass
TUG: Timed Up and Go
BMI: Body Mass Index
BW: Body Weight
